# Effects of Chicory (*Cichorium intybus* L.) Extract on Male Rat Reproductive System, Pregnancy and Offspring Development

**DOI:** 10.3390/ph17060700

**Published:** 2024-05-28

**Authors:** Alexandra N. Babenko, Lubov V. Krepkova, Marina V. Borovkova, Olga S. Kuzina, Vladimir A. Mkhitarov, Kathleen M. Job, Elena Y. Enioutina

**Affiliations:** 1 All-Russian Institute of Medicinal and Aromatic Plants (VILAR), Moscow 113628, Russia; alexandra.mogileva@gmail.com (A.N.B.); krepkowa2011@yandex.ru (L.V.K.); borovkova_65@mail.ru (M.V.B.); oskt@list.ru (O.S.K.); 2 FSBI “Research Institute of Human Morphology”, Moscow 117418, Russia; mkhitarov@mail.ru; 3 Division of Clinical Pharmacology, Department of Pediatrics, School of Medicine, University of Utah, Salt Lake City, UT 84108, USA; kate.job@hsc.utah.edu

**Keywords:** chicory extract, male reproductive system, spermatogenesis, fertility, offspring development

## Abstract

Background: We recently reported that extract prepared from the aerial part of *Cichorium intybus* L. (CE) possesses hepatoprotective, hypolipidemic, and hypoglycemic properties. This paper focuses on the effects of CE on the male rat reproductive system and the effects of this treatment on pregnancy and offspring development. Methods: The experimental male rats received 100 mg/kg bw/day, 500 mg/kg bw/day, and 1000 mg/kg bw/day of CE orally for 60 consecutive days. Rats that received tap water were used as controls. After treatment, we evaluated the effects of CE on the male reproductive system, fertility, and offspring development. Results: For CE-treated male rats, there was a significant increase in the (1) diameter of seminiferous tubules, (2) spermatogenic index, (3) number of total and motile spermatozoa, and (4) testosterone levels. Additionally, there was a decrease in the pre- and post-implantation death of the embryos in the CE-treated group. All pups born from CE-treated males demonstrated normal development. Conclusions: CE treatment significantly improved male reproductive functions. No adverse effects on pregnancy and offspring development were observed when males were treated with CE. Further clinical evaluation of CE should lead to the development of a safe and effective phytodrug for treating male infertility.

## 1. Introduction

Over the past several decades, the total fertility rates in Europe, North America, and Australia dropped below the rate necessary to maintain the population rate [1,2,3]. Reasons for decreasing fertility rates is the growing number of both male and female infertility cases. About one-third of infertility cases were associated with changes in the male reproductive system, and an additional 30% were related to problems in male and female reproductive systems [3]. The increase in infertility is likely due to lifestyle changes (e.g., reduced sleep time, dietary changes, use of alcohol, tobacco, and narcotics), poor health conditions (e.g., obesity), or environmental pollution (e.g., industrial chemical exposure, heavy household chemical use) [2,4]. These reasons for infertility are unlikely to change in the near future. Therefore, medical interventions are being pursued to return the fertility rates to maintenance levels.

Several pharmacological and microsurgical methods are available for the treatment of male infertility [5]. Treatments include microsurgical testicular sperm extraction, hormonal therapy (e.g., the use of gonadotropins and selective estrogen receptor modulators), and the use of aromatase inhibitors [5].

Many patients, however, prefer to use traditional methods of treatment, including the use of acupuncture and phytodrugs. Multiple studies have presented the positive effects of various medicinal plants or extracts prepared from them on the male reproductive system. Medicinal plants such as Tribulus (*Tribulus terrestris*), bananas (e.g., *Musa acuminata*), nigella (*Nigella sativa*), burdock (*Arctium lappa*), and fenugreek *Trigonella foenum-graecum* may stimulate spermatogenesis [6,7]. There are also reports that the use of *Panax ginseng* C.A.Mey, *Dáucus caróta*, *Passiflora incarnata*, *Rhaponticum carthamoides* (Willd.), *Allium cepa* L., *Állium satívum*, *Camellia sinensis*, *Amaranthus spinosus*, *Lepidium meyenii*, *Morinda officinalis*, *Schisandrae chinensidis* plant, root or seed extracts may improve semen parameters, hormonal levels, and sexual dysfunctions in both humans and experimental animals [7,8,9,10,11,12].

Recently, we conducted a preclinical evaluation of a highly purified extract prepared from the aerial part of wild chicory (*Cichorium intybus* L., CE). The extract demonstrated hepatoprotective, hypolipidemic, and hypoglycemic effects [13].

Our present studies have evaluated the impact of CE treatment on male rats’ reproductive functions and fertility. We determined that treatment with CE improved the male rat reproductive system functions, evidenced by an increase in the diameter of seminiferous tubules, spermatogenic index, number of total and motile spermatozoa, and testosterone levels. Another research group has reported that the treatment of male rats with a different extract prepared from chicory leaves increased the weight of testes and epididymis, stimulated the production of morphologically normal sperm, and increased serum testosterone levels [14]. The authors attributed the gonadotropic effects to the antioxidant properties of the bioactive components present in the extract. While the extracts were prepared using different extraction methods and the extracts’ compositions can differ significantly, the findings show that extracts prepared from the aerial part of chicory may stimulate spermatogenesis in male rats.

To the best of our knowledge, there were no reported studies investigating the effects of the phytodrug impacting male reproductive function on pregnancy or postnatal offspring development. This study additionally examines the impact of CE treatment of male rats on pregnancy and offspring development. The study results show that male rat treatment with CE did not affect pregnancy and offspring development.

## 2. Results

As part of the CE preclinical pharmacological and safety studies, we have investigated its effects on the reproductive function of male rats, fertility, and effects on offspring development.

### 2.1. The Effect of CE on the Reproductive Function of Male Rats

One of the most critical indicators that characterize the testes’ morphological state is its relative mass. It is defined as the ratio of organ mass to body mass, expressed as a percentage. The administration of CE for 60 consecutive days to male rats in doses of 100, 500, and 1000 mg/kg bw/day had no significant effect on relative testicular weight (Table 1).

**Figure 1:**
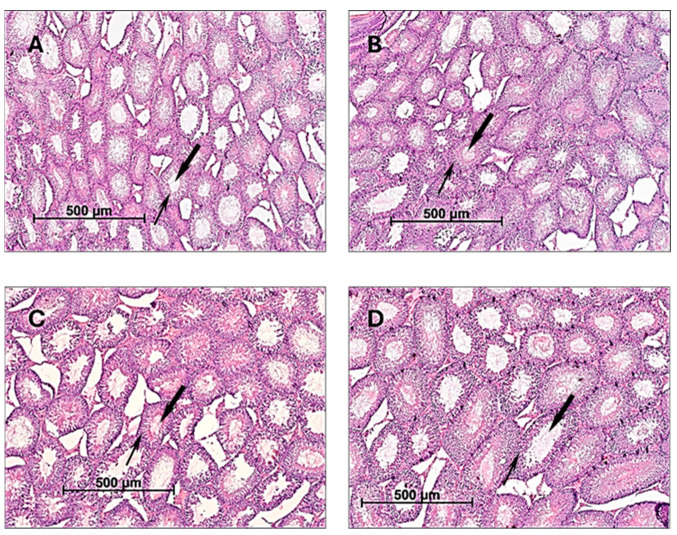
The morphological structure of testes of male rats. Male rats were orally treated for 60 days with (**A**)—H_2_O (control); (**B**)—CE 100 mg/kg bw/day; (**C**)—500 mg/kg bw/day; and (**D**)—1000 mg/kg bw/day. Thin arrow—seminiferous spermatogenic epithelium of the testes; bold arrow—lumens of the seminiferous tubule. Staining hematoxylin/eosin; magnification ×50.

There is evidence of active spermatogenesis at different stages of development in control and experimental groups. The morphological structure of the testes of male rats receiving CE at 100, 500, or 1000 mg/kg bw/day for 60 consecutive days appeared normal and was not different from the control group (Figure 1). The testes have a dense connective tissue membrane, from which connective tissue septa extend to the center. The septa contain numerous elastic fibers dividing the organ into lobules. The inner side of the seminiferous tubules is covered with spermatogenic epithelium, which contains morphologically normal spermatogenic cells.

**Figure 2:**
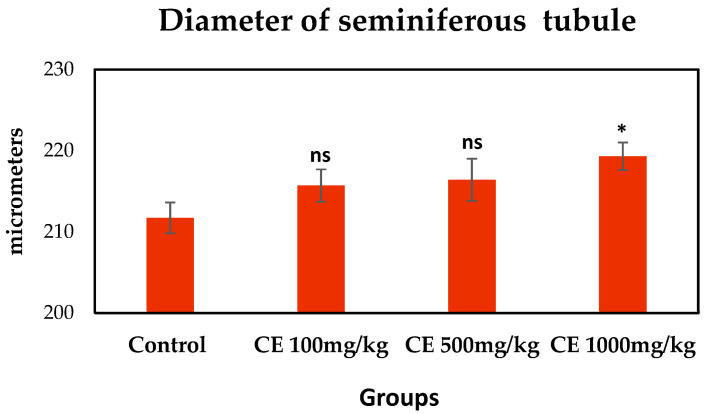
Effect of CE treatment on the diameter of seminiferous tubules. Control rats were orally treated with H_2_O for 60 days; experimental rats received 100, 500, and 1000 mg/kg bw/day of CE by gavage for 60 days. *—differences are statistically different compared to control (*p* = 0.0339). ^ns^—the differences between the CE-treated and control groups were not statistically significant.

The productive function of the testes was investigated based on three parameters: the diameter and cross-sectional area of the convoluted seminiferous tubules and the spermatogenesis index. The highest dose CE (1000 mg/kg bw/day) treatment significantly increased the diameter of the seminiferous tubules in the rats (Figure 2) when compared to the control group (*p* = 0.0339).

**Figure 3:**
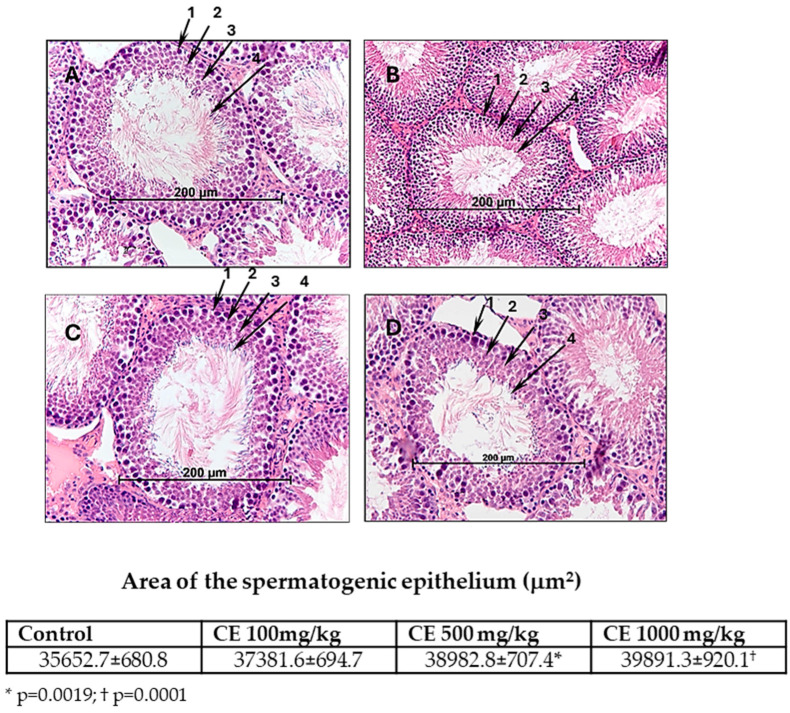
The effects of CE treatment on spermatogenic epithelia. Male rats were orally treated for 60 days with (**A**)—H_2_O (control); (**B**)—CE 100 mg/kg bw/day; (**C**)—500 mg/kg bw/day; and (**D**)—1000 mg/kg bw/day 1—spermatogonia; 2—spermatocytes; 3—spermatids; 4—spermatozoa. Staining hematoxylin/eosin; magnification ×200.

CE treatment increased the total area of the spermatogenic epithelium compared to control; however, statistically significant differences were observed only in doses of 500 (*p* = 0.0019) and 1000 mg/kg bw/day (*p* = 0.0001) (Figure 3). Representative morphological structures of spermatogenic epithelia are presented in Figure 3.

The spermatogenesis index is an essential marker of the testes’ reproductive function and demonstrates the spermatogenic epithelium’s functional activity. The oral administration of CE in doses 500 and 1000 mg/kg bw/day stimulated spermatogenesis in male rat testes; the differences were statistically significant (*p* = 0.001–0.002, Table 2).

### 2.2. The Effect of CE on Morpho-Functional Parameters of Epididymal Spermatozoa of Male Rats

The condition of spermatozoa was assessed by counting the total and motile number of spermatozoa, the duration of movement, and the number of pathological forms. The oral administration of CE (100, 500, and 1000 mg/kg bw/day) for 60 consecutive days to male rats had a significant positive effect on the main morpho-functional parameters of spermatozoa (Table 3). The CE treatment increased the total number of spermatozoa. Differences between the CE-treated and control groups were statistically significant (*p* < 0.05). The number of motile spermatozoa in the homogenate of the epididymis from CE-treated groups has been increased compared to those in the control group. The duration of movement and the number of pathological forms of spermatozoa in CE-treated groups were not different from those in the control group. These results indicate that CE at the tested doses had a stimulating effect on spermatogenesis.

### 2.3. Characteristics of the Functional State of Leydig Cells

**Figure 4:**
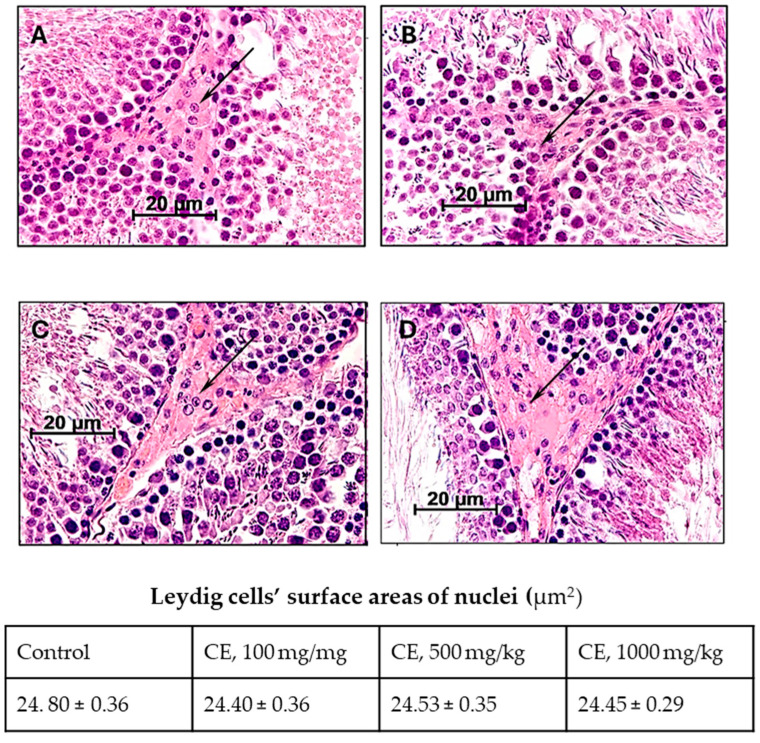
The effects of CE treatment on Leydig cells. Male rats were orally treated for 60 days with (**A**)—H_2_O (control); (**B**)—CE 100 mg/kg bw/day; (**C**)—500 mg/kg bw/day; and (**D**)—1000 mg/kg bw/day. Arrows—Leydig cells. Staining hematoxylin/eosin; magnification × 640.

Testosterone is one of the most essential androgenic hormones that regulate fertility, development, and maintenance of the male reproductive system and sexual function [15]. Testosterone is secreted by endocrinocytes, also called Leydig cells, located in the interstitial connective tissue of the testes. One of the indicators of the functional activity of Leydig cells is the surface area of their nuclei. The treatment of male rats with CE did not increase the surface areas of nuclei compared with the control group (Figure 4)**.**

**Figure 5:**
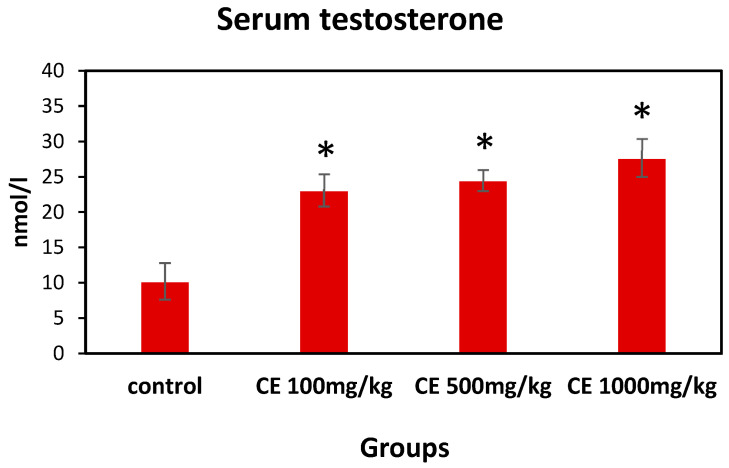
The effect of CE treatment on serum testosterone levels. Control rats were orally treated with H_2_O for 60 days; experimental rats received 100, 500, and 1000 mg/kg bw/day of CE by gavage for 60 days. The serum total testosterone concentrations were measured on day 60 of treatment. *—*p* < 0.05.

Serum testosterone concentrations were significantly higher in the animals receiving CE at all tested doses than in control animals (Figure 5). These results may suggest an increase in the level of sexual activity and fertility of males treated with CE.

### 2.4. Effects of CE on Male Rat Fertility

The fertility indicator is an integral criterion of the effectiveness of spermatogenesis and its sensitivity to harmful factors. The fertility of experimental animals was determined by mating intact female rats with control or CE-treated mature male rats. Subsequently, the fertility and pregnancy index was calculated.

The oral CE administration (100, 500, and 1000 mg/kg bw/day) to male rats for 60 days before mating with intact females did not affect the body weight dynamics of pregnant female rats after their impregnation. The fertility index was consistent between groups of female rats mated with CE-treated males and female rats mated with control males (Table 4). All females mated with CE-treated or control male rats became pregnant (Table 4).

The effects of CE treatment of male rats before mating on pre- and post-implantation embryonic death were investigated on day 20 of pregnancy (Table 5). CE treatment did not significantly change embryonic pre- and post-implantation death compared to the control group. However, pregnant females who were mated with males treated with 500 mg/mL and 1000 mg/mL tended to have less embryonic pre- and post-implantation death. Fetal weight on day 20 of pregnancy was similar in all treatment groups compared to the control group (Table 5).

### 2.5. The Effect of CE Male Rat Treatment on Offspring Development

The parameters for postnatal offspring development (the number of newborns in litters, the number of surviving pups, and their body weights in all experimental groups) in CE-male-treated groups were not statistically different compared to the control group (Table 6). However, the number of newborn pups was slightly higher in the groups born from male rats treated with 500 mg/kg bw/day and 1000 mg/kg bw/day. The number of surviving pups in the groups born from male rats treated with 500 mg/kg bw/day and 1000 mg/kg bw/day was higher compared to the control group, and all pups survived in the group born from male rats treated with 1000 mg/kg bw/day.

The physical development (e.g., pinna detachment, fur appearance, and eye-opening) in pups of all experimental groups was similar to the pups in the control group and corresponded to the physiological norm. There were no statistically significant differences in the negative geotaxis skills of experimental rat pups compared to the control animals (Table 7). The negative geotaxis test examines the maturity of motor skills and cerebellar integration in pups [16,17]. While there were no statistically significant differences in the open-field test between pups born from rat males treated with CE and control pups, the pups born from males treated with CE were more active, as evidenced by increased numbers of square crossing, head dipping and their emotional status (i.e., frequency of defecation) (Table 7).

## 3. Discussion

Multiple studies have reported the enhancement of spermatogenesis by phytodrugs or extracts from medicinal plants, including extracts from various species of chicory [6,7,8,12,14,18,19,20]. One difficulty in comparing the pharmacological effects of phytodrugs or extracts obtained from the same medicinal herb is that the studies may use extracts from different plant parts (e.g., whole plant, aerial part of the plant, or roots). The composition of the biologically active constituents in various plant parts varies significantly. For example, the HPLC fingerprinting analysis of different parts of chicory (e.g., flowers, stems, leaves, and roots) established that the highest amount of bioactive constituents is present in flowers, followed by stems, leaves, roots, and seeds [21]. The leaves and stems had the highest amounts of cichoric acid, flowers had the highest levels of esculin, and roots were rich in chlorogenic acid. Additionally, the extract composition may differ significantly depending on the extraction methods (e.g., water, ethanol, or a mixture of water and ethanol).

One of the first publications by Roy-Choudhury A. and Venkatakrishna-Bhatt H. on the effects of chicory extracts on reproductive functions of male animals showed that *Cichorium intybus* L. “aqueous root suspension” suppressed spermatogenesis in mice [22]. Our data demonstrate that the CE treatment improved the reproductive function of male rats. CE was prepared from the *Cichorium intybus* L. aerial part, which included stems, leaves, and flowers. The therapy with CE increased spermatogenesis and testosterone levels in healthy male rats. Another publication also showed chicory extract’s stimulatory effects on the male rats’ reproductive system [14]. The extract was prepared from the dried *Cichorium intybus* L. leaves by extraction of bioactive compounds with ethanol. The extract was orally administered to healthy adult male rats at doses 50, 100, and 200 mg/kg daily for 70 consecutive days. Unlike in our study, the chicory ethanolic extract administration significantly increased the testicular and epididymal weights. The authors reported a statistically significant increase in “sperm density” and a reduction in morphologically abnormal forms of spermatozoa. Spermatozoa motility in extract-treated rats were similar to the control group. Our study found an increase in the total number of spermatozoa and motile forms. Both studies showed an increase in testosterone levels. Different bioactive molecules in the extracts could explain the observed difference in the effects of chicory extract obtained by ethanol extraction and CE on the male reproductive system. Unfortunately, we could not find more details on the extraction method and extract’s chemical composition. The mechanism of action of the chicory ethanolic extract was potentially associated with the extract’s antioxidant properties [14].

Koloko B. et al. investigated the effect of *Rauvolfia vomitoria* ethanolic extract on reproductive activity and sexual performance of healthy male rats [20]. Rats were treated with the extract at doses of 50, 100, and 200 mg/kg bw/day for 22 days. The *Rauvolfia* extract increased the spermatozoa count and their motility. *Rauvolfia* extract-treated male rats demonstrated an increase in sexual activity. The mount latency, intromission latency, and post-ejaculatory intervals were significantly reduced, while ejaculation latency, mount frequency, intromission frequency, and ejaculation frequency were significantly increased compared to control animals.

Exposure to metal particles (e.g., lead, cadmium, aluminum, arsenic, and chromium) through air pollution may lead to male infertility [23]. Lead is a well-known environmental pollutant. Lead significantly decreased the spermatozoa concentrations and the number of motile spermatozoa in male rats [18]. Co-administration of chicory extract with lead restored the number of motile spermatozoa to control group levels and reduced the number of abnormal forms. Humans are frequently exposed to aluminum particles through air pollution, antiperspirants, cooking containers, canned food, and cosmetics. Aluminum exposure may result in damaging effects on the male reproductive system [24]. The exposure of male rats to aluminum chloride at a dose of 50 mg/kg bw/day for 70 days resulted in decreased spermatozoa count and motility and an increase in dead cells and morphologically abnormal forms of spermatozoa. Treatment with esculetin, a coumarin derivative, ameliorated adverse aluminum chloride effects on the male rat reproductive system.

Tobacco smoke is another air pollutant that produces fine particles. Hashemi M. et al. evaluated the effect of 70% ethanol/water chicory extract on the reproductive function of male rats exposed to cigarette smoke [25]. The treatment with the chicory extract reduced damage from cigarette smoke to male rat reproductive functions. The chicory treatment of rats exposed to smoke increased the number of Sertoli cells, spermatogonia, and spermatocytes and reduced the number of apoptotic cells otherwise damaged by exposure to smoke.

Certain drugs may have adverse effects on the male reproductive system. It has been reported that aldosterone inhibits stem Leydig cell proliferation, while dexamethasone inhibits their differentiation [26]. Animal studies determined that prenatal exposure to dexamethasone leads to spermatozoa quality in male offspring [27]. It has been proposed that these changes are associated with epigenetic programming of the Sertoli cells. *Portulaca oleracea* and *Cichorium intybus* extracts synergistically enhanced male rat fertility in the dexamethasone-induced testicular and autophagy dysfunction model. The treatment with these extracts of dexamethasone-exposed male rats increased sperm motility and showed a protective effect on seminiferous tubules. The potential mechanism of action of the *Portulaca oleracea* and *Cichorium intybus* is proposed through attenuating oxidative stress and autophagy. *Ocimum tenuiflorum*, a medicinal plant with antioxidant properties, protected spermatozoa and germ cells from filgrastim damaging effects [28].

Excessive oxidative stress is one of the mechanisms responsible for male infertility [29,30,31]. It damages spermatozoan activities and male fertility. Reactive oxygen species (ROS) are produced during the maturation and capacitation of spermatozoa, as well as by the prostate and seminal vesicles. Excessive ROS production may reduce sperm motility and viability and damage DNA and other cellular components. Chronic inflammation also plays a significant role in male infertility. Mehran Dorostghoal and colleagues investigated the mechanisms of the gonadotropic effect of the chicory leaf extract. They determined that the extract reduced malondialdehyde levels and increased superoxide dismutase and glutathione peroxidase activity in the testicles of treated rats [14]. The authors suggested that the improvements in male reproductive health may be associated with the extract’s antioxidant and androgenic properties. Other investigators agree that the antioxidant and anti-inflammatory properties of medicinal plants such as cinnamon (*Cinnamomum verum*), grape (*Vitis vinifera*), onion (*Allium cepa*), garlic (*Allium sativum*), apricot (*Prunus armeniaca*), dogwood (*Cornus florida*) are responsible for the improve sperm quality and the number of motile sperm in patients with asthenozoospermia [6]. The modulations of extracellular-regulated kinase (ERK), protein kinase B (PKB), and NF-kB signaling pathways by the phytodrugs are mechanisms regulating male fertility [8]. Another potential mechanism responsible for the anti-infertility properties of the phytodrugs is smooth muscle-relaxing properties, which could help reduce male infertility [31].

Most of the above-presented studies focused on the effects of herbal extracts on the male reproductive system. Occasionally, medicinal herbs and phytodrugs may represent a potential risk to the childbearing mother, the fetuses, and offspring born from mother or father exposed to herbal remedies. Our study also demonstrated that male exposure to CE did not affect pregnancy development; CE male treatment reduced pre- and post-implantation embryonic death. The systematic review investigating the effect of an oral antioxidant, *Satureja khuzestanica,* on male infertility and pregnancy rate concluded that the plant increases spermatozoa quality and pregnancy rate [32]. The bioflavonoid composition from the roots of *Scutellaria baicalensis* and the heartwoods of *Acacia catechu* administered to either male rats for 4 weeks or female rats for 2 weeks before mating at the highest dose of 1000 mg/kg had no significant effects on male fertility indexes, but it appears that increased implantation rate and reduced embryo mortality [33].

Our current study also reported that CE treatment of male rats had no significant effect on offspring development. However, there is a limitation to this study. The International Standard OECD Test No. 416:2001 Two-Generation Reproduction Toxicity Study requires evaluation of reproductive toxicity on two generations of laboratory animals to provide information regarding the effects of the test substance on the development of F1 and F2 offspring. We assessed the CE effects on the growth and development of one F1 generation.

Animal studies demonstrate that herbal extracts have great potential to improve male reproductive health. Unfortunately, very few high-quality clinical trials were conducted to determine whether these herbal extracts can help to overcome male infertility in human patients. A meta-analysis of the effect of ginseng on erectile dysfunction analyzed data from randomized and semi-randomized trials [34]. The investigators concluded that the ginseng treatment had minimal adverse reactions; however, it had a “trivial effect” on erectile function, as evidenced by the measurements of the index of erectile function. Salgado R. et al. reported that *Tribulus terrestris* given to men with abnormal spermatogenesis increased dihydrotestosterone levels, enhanced sperm concentration, and motility [35]. Protodioscin, a bioactive *Tribulus* constituent, stimulated germ cell proliferation and growth of seminiferous tubules. Another review, however, concluded that *Tribulus terrestris* and *Lepidium meyenii* Walp treatment did not stimulate testosterone production in men [7].

Hsieh C. et al. investigated the impact of herbal formula as an adjunct therapy in males with impotence [36]. The herb formula was comprised of seven ingredients: *Astragalus membranaceus*, *Lepidium meyenii* Walp., *Ophiocordyceps sinensis*, *Panax quiquefolium* (American ginseng) 1, *Piper nigrum*, *Rhodiola rosea* and *Serpentes cnidium monnieri*. Males who had impotence and were refractory to conventional drug treatment underwent penile venous stripping surgery, an effective technique for the treatment of impotence. Those patients who were unsatisfied with the procedure results were randomly assigned to either an herbal formula or placebo treatment group. The combinational treatment of these patients improved the index of erectile function compared to the procedure alone.

A large body of preclinical evidence demonstrates the effectiveness of phytodrugs in improving animal reproductive functions. In our opinion, phytodrugs can be used as effective treatments for minor or moderate male reproductive functions in men. Additionally, the phytodrugs can be used as an adjunct therapy in severe cases of reproductive system dysfunctions.

Metabolic syndrome is a common disorder in men. The syndrome is presented with hypertension, dyslipidemia, and altered glucose metabolism. Smoking, excessive alcohol use, and limited physical activity are shared risk factors for the development of metabolic syndrome and male infertility [37]. The advantage of CE use is that CE targets multiple organs and systems. It possesses hepatoprotective, hypolipidemic, hypoglycemic, and male reproductive system-enhancing properties [13,38]. Treatment with CE can benefit patients with metabolic syndrome and developing infertility.

CE and other phytodrugs enhancing male fertility may be of interest to andrologists and patients as a mono or adjuvant therapy. More high-quality clinical trials are needed to introduce these phytodrugs to clinical practice.

## 4. Materials and Methods

### 4.1. Phytodrug

During the flowering period, the aerial part of wild chicory (*Cichorium intybus* L.) was collected and used to prepare the dry extract (CE) used in this study. CE was developed at the Institute of Medicinal and Aromatic Plants, VILAR. The plant material was harvested from 2020 to 2021 in the Ryazan region. CE was obtained by extracting bioactive constituencies with 70% *v*/*v* ethyl alcohol at 50 ± 5 °C. CE was standardized by the phenolic constituents calculated as chicoric acid. The extract used in the current study contained 9.20 + 0.46% *w/w* of phenolic constituents. Additional details on the extract preparation and chemical analysis can be found in the recently published article by Krepkova et al. [13].

### 4.2. Experimental Animals

CE effects on the male reproductive system, male fertility, and offspring development were investigated in Wistar rats obtained from the animal nursery “Andreevka”, Moscow, Russia. Three-to-four-month-old females (180–200 g) and males (200–250 g) were used in the study. Animals were housed in plastic cages with 4–5 rats per cage. Animals were kept under controlled environmental conditions of light (12 h light cycle), temperature (20–22 °C), and humidity (40–60%) with free access to Laboratorkorm standard chow for laboratory rats and tap water. After two weeks of acclimation, rats were used in the study. Following short procedures, rats were lightly anesthetized with isoflurane using a drop jar method to minimize unnecessary stress. At the end of each experiment, adult animals and offspring were euthanized by CO_2_ inhalation. Feti were considered to be euthanized after the confirmed death of the mother.

All studies were conducted according to the “Guidelines for conducting preclinical studies of drugs” implemented by the Ministry of Public Health of the Russian Federation [39]. The study protocols No 42 from 2 May 2021, No 43 from 3 March 2021, and No 59 from 11 August 2021 had been approved by the VILAR Ethical Committee on Animal Experimentation and carried out per the “European Convention for the Protection of Vertebrate Animals Used for Experimental and Other Scientific Purposes (ETS 123). Strasbourg, 1986” [40]. The numbers of experimental animals were chosen in accordance with the National Standard of the Russian Federation (GOST 56698-2015), identical to the International Guidelines OECD Test No. 416:2001 Two-Generation Reproduction Toxicity Study, IDT.

### 4.3. CE Treatment

Male rats were randomly assigned into four groups to evaluate the effects of CE on the male reproductive system, fertility, and offspring development. Rats were lightly anesthetized before CE or water administration by gavage. Control animals (18 per group) received tap water by gavage for 60 consecutive days. The experimental rats (18 per group) received 100 mg/kg bw/day, 500 mg/kg bw/day, and 1000 mg/kg bw/day of the 10% aqueous solution of CE orally for 60 consecutive days. The 10% aqueous CE solution was prepared daily. The experimental doses were chosen based on the pharmacological activity of the drug (hepatoprotective activity) and the evaluation of the safety profile of phytodrug in chronic experiments. It has been determined that the hepatoprotective therapeutic daily dose in rats was 100 mg/kg bw/day [13,41]. The doses exceeding the therapeutic dose 5- and 10-fold (500 and 1000 mg/kg bw/day, respectively) were well tolerated by rats in chronic experiments. Therefore, we have chosen these doses to evaluate the effects of CE on reproductive functions. The amount of the drug was calculated for each animal based on the body weight determined at the last weighing.

### 4.4. Analysis of CE Effects on the Male Reproductive System

Evaluation of the gonadotropic effects of CE was conducted the next day after treatment completion (day 61). Sperm was obtained from 10 male rats in each group by vortexing a longitudinally cut-open epididymis in 10 mL of 0.9% sodium chloride for two minutes. At least 200 spermatozoa per animal were counted to enumerate total and motile spermatozoon numbers. Sperm motility was presented as a percentage of motile sperm in a sample. The sperm motility time was determined by placing a drop of sperm suspension onto a slide in a humid chamber at ~37 °C. Sperm motility was monitored every 5–7 min until complete cessation of all sperm movement.

Another drop of sperm suspension was placed onto the histological slide, fixed with ethanol for 10 min, air-dried, and stained with Giemsa staining to assess the presence of pathologic forms of spermatozoa. The fixed slides were reviewed under a microscope (magnification ×200–400). A minimum of five to seven view fields was counted.

The testes’ absolute and relative weights were initially determined following morphometric analysis of the testes. Then, ten-millimeter testicular pieces were fixed in 10% neutral formalin, embedded in paraffin, and sectioned to assess the testicular morphology. Sections of 10 μm were stained with hematoxylin and eosin. The sections were microfilmed on an Axioplan 2 Imaging Zeiss microscope equipped with an AxioCam HRc video camera at various magnifications with a 2776 × 2080 pixels/inch resolution. All measurements described below were conducted using the Image-Pro Plus 6.0 program (Media Cybernetics, Rockville, MD, USA), the “Measurement” function.

The tubules’ diameter was assessed at a magnification of ×50. Only the diameters of tubules cut tangentially were measured: 20 tubules per animal and 100 per group.

Twenty tangential sections of the seminiferous tubules were evaluated at a magnification of x200 to calculate the area of the spermatogenic epithelium. The area of the seminiferous tubule and lumen in µm^2^ was measured. The area of the spermatogenic epithelium was calculated as the area of the seminiferous tubule minus the luminal area.

The microfilms of twenty endocrinocytes (Leydig cells) per group were evaluated at a magnification of ×640 to measure the surface nuclear areas of Leydig cells. The surface nuclear areas were measured by manual nucleus contouring.

The spermatogenesis index was used to quantitatively assess the spermatogenic competence of the testes. The number of spermatogenic epithelium layers was counted in 100 convoluted seminiferous tubules per group. The index of spermatogenesis was calculated using the formula I = ∑ n/N, where n is the number of layers of spermatogenic epithelium (spermatogonia, spermatocytes, spermatids, and spermatozoa) in one tubule, N is the number of tubules examined.

The total testosterone concentrations in male rats’ serum were measured using an enzyme immunoassay using ImmunoChem-2100 (High Technology, Inc., North Attleborough, MA, USA) and a set of reagents (ZAO NVO Immunotech, Moscow, Russia).

### 4.5. Analysis of CE Effects on Male Fertility

In total, 15 intact females were mated with 7–8 males CE treated or control males. The effects of CE treatment on male fertility were evaluated by measuring fertility and pregnancy indexes. The fertility index was calculated as follows: the number of pregnant rats divided by the number of fertilized rats multiplied by 100. The pregnancy index was calculated as a ratio between the number of delivered dams and the number of pregnant dams multiplied by 100.

### 4.6. Investigation of PADE Effects on Fetal and Offspring Development

Pregnant rats were divided into two cohorts. Female rats from Cohort 1 were used to investigate CE effects on fetal growth, and female rats from Cohort 2 were used to analyze the impact of CE male treatment on offspring development. Pregnant rats of Cohort 1 (7 dams in each group) were sacrificed on day 20 of pregnancy by carbon dioxide asphyxiation. The number of corpora lutea, the status of each implant site (live/dead embryo, early/late resorption), and embryo body weight were determined. Cohort 2 (7 dams in each group) delivered their offspring by natural route. Weight gain and survival of the born offspring were assessed and recorded on day 21 after birth. Pups (47–51 in each group) were evaluated for reflex maturity: flip/roll over onto all four paws (day 7 after birth), time of cliff avoidance (day 7 after birth), and negative geotaxis (day 8 after birth). The development of motor-sensory reflexes of the progeny (open field test) was evaluated on day 30 after birth. The open field apparatus consisted of an unobstructed field with walls preventing animal escape. The field is marked with black and white squares. The corners of each square had round holes. Each test animal was placed into the field for 3 min. An investigator recorded the number of lines crossed by the rat and how often the animal was rearing, grooming, defecating, and head dipping into the holes. The flip/rollover test measured the percentage of pups rolling onto their four paws within 30 s. The negative geotaxis test measured motor skills and cerebellar integration. Pups were placed on an inclined surface (45° angle, length 30 cm) with their heads down. The time (s) spent for a turn of 180° was recorded.

### 4.7. Statistical Analysis

All data obtained from experimental or control rats were included in the analysis. Results included male fertility, gonadotropic effects, pre- and post-implantation death, number of newborns, and offspring postnatal development. The results were analyzed using Statistica software, version 13 (TIBCO Software Inc., Santa Clara, CA, USA). One-way ANOVA, Student’s *t*-test of significance, and test of the equality of continuous (the Kolmogorov–Smirnov test) were used for data analysis. The differences were considered statistically significant when the *p*-value was less than 0.05.

## 5. Conclusions

Preclinical studies presented in this manuscript demonstrated that CE has significant stimulatory effects on the male reproductive system, evidenced by an increase in the diameter of seminiferous tubules, spermatogenesis index, total number of spermatozoa and their viable forms, and serum testosterone levels. This study also evaluated the effect of CE treatment of male rats on fertility, pregnancy, and offspring development. No adverse effects were observed on pregnancy, evidenced by no change in the fertility and pregnancy indexes and embryonic pre- and post-implantation death. There was a nonsignificant decrease in pre- and post-implantation fetal death in groups where males were treated with higher doses of CE. The offspring development of pups born from CE-treated males was not different from control pups.

## Figures and Tables

**Table 1:** The effect of CE on the weight of testes of male rats.

Parameters	Experimental Groups ^a^			
	Control (H_2_O)	CE 100 mg/kg bw/day	CE 500 mg/kg bw/day	CE 1000 mg/kg bw/day
Number of animals evaluated	10	10	10	10
Weight of testes (g) ^b^	3.21 ± 0.20	3.20 ± 0.21 ^ns^	3.20 ± 0.07 ^ns^	3.20 ± 0.14 ^ns^
Relative weight (%) ^b^	0.96 ± 0.04	0.96 ± 0.04 ^ns^	0.94 ± 0.09 ^ns^	0.98 ± 0.08 ^ns^

^a^—Male rats were treated with either H_2_O (control) or CE (100, 500, or 1000 mg/kg bw/day) for 60 days before mating; ^b^—mean ± SE. ^ns^—the differences between the CE-treated and control groups were not statistically significant.

**Table 2:** The CE effect on the spermatogenesis index.

Parameters	Experimental Groups ^a^			
	Control (H_2_O)	CE 100 mg/kg bw/day	CE 500 mg/kg bw/day	CE 1000 mg/kg bw/day
Number of animals evaluated	10	10	10	10
Spermatogenesis index ^b^	3.47 ± 0.02	3.49 ± 0.02 ^ns^	3.71 ± 0.02 *	3.76 ± 0.02 **

^a^—Male rats were orally treated with either H_2_O (control) or CE (100, 500, or 1000 mg/kg bw/day) for 60 days; ^b^—mean ± SE. * *p*  =  0.001, ** *p* = 0.002; ^ns^—the differences between the CE-treated and control groups were not statistically significant.

**Table 3:** The CE effect on the spermatogenesis of male rats.

Parameters	Groups ^a^			
	Control (H_2_O)	CE 100 mg/kg bw/day	CE 500 mg/kg bw/day	CE 1000 mg/kg bw/day
The number of animals evaluated	10	10	10	10
Total number of spermatozoa (×10^6^) ^b^	23.5 ± 2.4	36.3 ± 3.3 *	37.4 ± 3.6 *	40.0 ± 4.6 *
Motile spermatozoa (×10^6^) ^b^	17.2 ± 1.7	23.7 ± 2.4 *	23.8 ± 2.2 *	32.9 ± 2.6 *
Duration of spermatozoon movement (min.) ^b^	360.0 ± 23.5	362.2 ± 26.0 ^ns^	345.3 ± 25.6 ^ns^	352.7 ± 23.5 ^ns^
Pathologic forms of spermatozoa (%)	1.5 ± 0.2	1.7 ± 0.2 ^ns^	1.6 ± 0.1 ^ns^	1.7 ± 0.2 ^ns^

^a^—male rats were orally treated with either H_2_O (control) or CE (100, 500, or 1000 mg/kg bw/day) for 60 days before mating; ^b^—mean ± SE. * *p*  < 0.05, ^ns^—the differences between the CE-treated and control groups were not statistically significant.

**Table 4:** Effects of CE on male rat fertility.

Parameters	Experimental Groups ^a^			
	♂ × ♀	♂ × ♀	♂ × ♀	♂ × ♀
	H_2_O × Intact	CE, 100 mg/kg × Intact	CE, 500 mg/kg × Intact	CE, 1000 mg/kg × Intact
Number of mated females	15	15	15	15
Number of fertile females	14	14	14	14
Number of pregnant females	14	14	14	14
Fertility index (%)	93.3	93.3	93.3	93.3
Pregnancy index (%)	100	100	100	100

^a^—male rats were orally treated with either H_2_O (control) or CE (100, 500, or 1000 mg/kg bw/day) for 60 days before mating.

**Table 5:** Effect of CE treatment of male rates on embryonic pre- and post-implantation death of day 20 of pregnancy.

Parameters		Experimental Groups ^a^			
		♂ × ♀	♂ × ♀	♂ × ♀	♂ × ♀
		H_2_O × Intact	CE, 100 mg/kg × Intact	CE, 500 mg/kg × Intact	CE, 1000 mg/kg × Intact
Number of pregnant female rats investigated		7	7	7	7
Embryonic Death (%) ^b^	Pre-implantation	7.7 ± 0.8	7.6 ± 0.7 ^ns^	7.2 ± 0.3 ^ns^	6.4 ± 0.6 ^ns^
	Post-implantation	4.7 ± 0.5	4.5 ± 0.3 ^ns^	4.2 ± 0.3 ^ns^	3.8 ± 0.4 ^ns^
Embryonic weight on day 20 (g) ^b^		2.4 ± 0.1	2.3 ± 0.1 ^ns^	2.4 ± 0.03 ^ns^	2.3 ± 0.1 ^ns^

^a^—male rats were orally treated with either H_2_O (control) or CE (100, 500, or 1000 mg/kg bw/day) for 60 days before mating; ^b^—mean ± SE. ^ns^—the differences between the CE-treated and control groups were not statistically significant.

**Table 6:** The effect of CE treatment of male rats on the postnatal offspring development.

Parameters			Experimental Groups ^a^			
			♂ × ♀	♂ × ♀	♂ × ♀	♂ × ♀
			H_2_O × Intact	CE, 100 mg/kg × Intact	CE, 500 mg/kg × Intact	CE, 1000 mg/kg × Intact
Number of litters evaluated			7	7	7	7
Number of newborn pups in the litter ^b^			8.4 ± 0.4	8.1 ± 0.7 ^ns^	8.5± 0.3 ^ns^	9.3 ± 0.8 ^ns^
Number of surviving pups per female rat post-delivery	Day 7	Number ^b^	8.1 ± 0.6	8.1 ± 0.7 ^ns^	8.5± 0.3 ^ns^	9.3 ± 0.8 ^ns^
		%	96.4	100	100	100
	Day 14	Number ^b^	8.1 ± 0.6	7.9 ± 0.6 ^ns^	8.5± 0.3 ^ns^	9.3 ± 0.8 ^ns^
		%	96.4	97.5	100	100
	Day 21	Number ^b^	7.9 ± 0.6	7.7 ± 0.6 ^ns^	8.3 ± 0.4 ^ns^	9.3 ± 0.8 ^ns^
		%	94.0	95.1	98.0	100
Pups weight (g) ^b^	Day 1		6.2 ± 0.2	6.3 ± 0.3 ^ns^	6.1± 0.4 ^ns^	6.0 ± 0.2 ^ns^
	Day 4		9.1 ± 0.5	9.0 ± 0.5 ^ns^	8.9 ± 0.7 ^ns^	8.9 ± 0.3 ^ns^
	Day 7		12.3 ± 0.8	12.7 ± 1.1 ^ns^	11.9 ± 0.5 ^ns^	11.8 ± 0.6 ^ns^
	Day 14		22.6 ± 0.9	22.9 ± 0.9 ^ns^	20.9 ±1.5 ^ns^	20.9 ± 1.3 ^ns^
	Day 21		32.4 ± 2.2	33.2± 2.0 ^ns^	32.2 ± 3.4 ^ns^	31.2 ± 3.0 ^ns^

^a^—male rats were orally treated with either H_2_O (control) or CE (100, 500, or 1000 mg/kg bw/day) for 60 days before mating; ^b^—mean ± SE. ^ns^—the differences between the CE-treated and control groups were not statistically significant.

**Table 7:** Postnatal offspring development.

Parameters				Groups ^a^			
				Control (H_2_O)	CE 100 mg/kg	CE 500 mg/kg	CE 1000 mg/kg
Number of pups evaluated				51	52	50	54
Time of cliff avoidance (sec.) ^b^				5.0 ± 0.6	4.7 ± 0.4 ^ns^	6.2 ± 0.5 ^ns^	5.3 ± 0.5 ^ns^
Flip/roll over onto all four paws		Pups with positive reaction	No.	51	52	50	54
			%	100	100	100	100
Negative geotaxis (sec) ^b^				11.3 ± 1.2	10.9 ± 1.1 ^ns^	11.5 ±1.3 ^ns^	10.9 ± 1.0 ^ns^
Open-field test	Total squares crossed ^b^			32.9 ± 3.2	37.6 ± 3.9 ^ns^	37.8 ± 4.0 ^ns^	38.0 ± 3.7 ^ns^
	Head dipping ^b^			3.17 ± 0.35	3.20 ± 0.33 ^ns^	3.50 ± 0.35 ^ns^	3.60 ± 0.35 ^ns^
	Grooming frequency ^b^			1.20 ± 0.13	1.20 ± 0.12 ^ns^	1.10 ± 0.11 ^ns^	1.10 ± 0.13 ^ns^
	Defecation ^b^			2.90 ± 0.22	3.10 ± 0.30 ^ns^	3.40 ± 0.34 ^ns^	3.60 ± 0.41 ^ns^
	Rearing frequency ^b^			0.10 ± 0.10	0.20 ± 0.13 ^ns^	0.20 ± 0.13 ^ns^	0.20 ± 0.13 ^ns^

^a^—male rats were orally treated with either H_2_O (control) or CE (100, 500, or 1000 mg/kg bw/day) for 60 days before mating; ^b^—mean ± SE. ^ns^—the differences between the CE-treated and control groups were not statistically significant.

## Data Availability

Data are available upon reasonable request.
